# Processing of Complex Auditory Patterns in Musicians and Nonmusicians

**DOI:** 10.1371/journal.pone.0021458

**Published:** 2011-07-07

**Authors:** Bastiaan Boh, Sibylle C. Herholz, Claudia Lappe, Christo Pantev

**Affiliations:** 1 Faculty of Psychology and Neuroscience, Maastricht University, Maastricht, The Netherlands; 2 Montreal Neurological Institute, McGill University, and International Laboratory for Brain, Music and Sound Research (BRAMS), Montreal, Canada; 3 Institute for Biomagnetism and Biosignalanalysis, Muenster, Germany; 4 Otto-Creutzfeldt-Center for Cognitive and Behavioral Neuroscience, Westfalian Wilhelms-University, Muenster, Germany; University of California, Irvine, United States of America

## Abstract

In the present study we investigated the capacity of the memory store underlying the mismatch negativity (MMN) response in musicians and nonmusicians for complex tone patterns. While previous studies have focused either on the kind of information that can be encoded or on the decay of the memory trace over time, we studied capacity in terms of the length of tone sequences, i.e., the number of individual tones that can be fully encoded and maintained. By means of magnetoencephalography (MEG) we recorded MMN responses to deviant tones that could occur at any position of standard tone patterns composed of four, six or eight tones during passive, distracted listening. Whereas there was a reliable MMN response to deviant tones in the four-tone pattern in both musicians and nonmusicians, only some individuals showed MMN responses to the longer patterns. This finding of a reliable capacity of the short-term auditory store underlying the MMN response is in line with estimates of a three to five item capacity of the short-term memory trace from behavioural studies, although pitch and contour complexity covaried with sequence length, which might have led to an understatement of the reported capacity. Whereas there was a tendency for an enhancement of the pattern MMN in musicians compared to nonmusicians, a strong advantage for musicians could be shown in an accompanying behavioural task of detecting the deviants while attending to the stimuli for all pattern lengths, indicating that long-term musical training differentially affects the memory capacity of auditory short-term memory for complex tone patterns with and without attention. Also, a left-hemispheric lateralization of MMN responses in the six-tone pattern suggests that additional networks that help structuring the patterns in the temporal domain might be recruited for demanding auditory processing in the pitch domain.

## Introduction

Listening or performing music engages the brain in many different ways involving learning, memory and emotions such as pleasure, causing music as a phenomenon to be a popular pastime. One important aspect of music processing is the ability to represent and categorize tone strings and recognize reoccurring patterns in the incoming information. This applies not only to music, but to auditory scene analysis in general, where pattern detection is an efficient way of reducing computation costs. When analysing the continuous auditory landscape around us, we are constantly moving from a holistic to a focal perspective, using attention to filter out what is then perceived as input that conflicts with the source of focus [1], so long as these sources stand out well enough to be recognized and ‘streamed’ independently [2]. Once a certain source is deemed of interest, either combinations of (adjacent) tones will be matched with possible pre-existing representations on-line dynamically [3], new representations will be formed after a certain reliable number of iterations (about three according to [4]), or existing representations will be updated with new sensory information [3], [5]. Any competing sound streams with similar content will be attenuated during this process [6]. In this respect, it is important to realize that with each new incoming tone, the existing representation of regularity or of a pattern can either be expanded, or altered by discarding its first tone. Importantly, representations of regularities can be formed and maintained on different hierarchical levels of complexity simultaneously [7].

Even in the absence of background noise, the generation of pattern representations is subject to how interpretable a continuous auditory stream is. Complex melodic sound sequences often consist of recurring sub-sections (or ‘units’), which are essentially small invariant patterns that can be detected and encoded (or: ‘chunked’) by the brain following heuristics of expectancy [8]. Such chunking increases general memory processing efficiency [9], enabling more elaborate features to be analyzed, and it is enhanced if sound strings exhibit Gestalt-esque features that enable them to be grouped in a meaningful way [10].

The level of detail on which a recurring pattern or regularity can be encoded differs across individuals, and seems to be enhanced by long- as well as short-term musical training. For example, musicians are able to encode more complex regularities in reoccurring tone sequences than nonmusicians [7], [11], [12] and short-term piano training enhances the ability to encode short melodies and detect incorrect tones [13]. Even so, due to music exposure in our everyday life, non-musicians have implicit knowledge about the context of tones and the expectancy associated with implicit heuristics on how patterns should be constructed, e.g. when its context is perceived as a ‘logical’ series of successive chords [14], [15]. Such contextual knowledge is structured into detailed representations of pitch relationships, which, for non-musicians can be on par with experts in music theory, especially for chords that are of the C-major scale [16], Non-musicians as well as experts build auditory expectancy models based on this contextual knowledge, wherein for example possible consecutive tones in a pattern (or melody) are each associated with a degree of expectancy based on previously perceived (and thus learned) relationships and occurrences [17], [18].

Unlike in visual processing, the external acoustic environment cannot be used as a sustained cue for reference. Instead, the brain has to make use of its internal storage and representation systems [19]. Most electrophysiological research on what such a system would entail concerning pattern processing has focussed on its automated, pre-attentive aspects (for a review, see [20]). The process of auditory pattern representation and maintenance on this pre-attentive level can (at least for a large part of it) be accounted for by applying the memory trace theory [21]. This theory states that networks in the auditory cortex are specialized in the pre-attentive detection of auditory regularity, both in music and in speech, generating a memory trace. This trace can then be used as input for a pre-attentive change-detection process that determines if new input is consistent or deviates from the expected. The memory trace fades over time and has been estimated to last around 10 seconds if not updated by new input [22], [23]. A common probe for the memory trace is a pre-attentive component of event-related potentials or fields, the mismatch negativity (MMN) that represents the detection of a deviance of a sound input from a regularity that has been encoded from the previous sounds [5], [20], [24]. This difference is commonly referred to as ‘deviant’ unique stimulation that follows a series of ‘standard’ repetitive stimulation. The MMN is visible as a deflection with the same polarity as the N100 component within an interval of approximately 110 to 250 ms after onset of the deviant sound.

Most research on the memory trace has either focused on sound properties that can be encoded (e.g., [11], [25], [26]), on the temporal window of integration that determines if sounds are encoded as individual units in the memory trace [27], or on the decay of the memory trace over time (e.g. most recently [28]). However, to our knowledge, the capacity of the memory trace in terms of how much information can be stored has not been systematically investigated, and not much is known about how long or complex sound patterns can be until a complete representation of a sound pattern can no longer be maintained. In the current study, these questions were investigated by utilizing the mismatch negativity in an MEG experimental setup. The MMN in response to deviants within repeated tone sequences consisting of different numbers of tones was used to indicate whether the overall tone pattern was sufficiently encoded in the auditory memory trace. Importantly, and in contrast to previous studies, deviants occurred in all positions of the pattern. Therefore, encoding of the whole pattern was necessary to detect all deviant tones. Furthermore, since advantages of music training have been found mainly for tasks that require quite specialized features to be detected, we hypothesized that musicians and non-musicians might differ in their ability to form representations of complex patterns of different lengths. By comparing musicians and nonmusicians we aimed at determining to what extent the capacity of the memory trace underlying the MMN is enhanced by long-term training.

Additionally, we have conducted in parallel a second experiment using a behavioural setup in which participants listened attentively to the identical (complex sequential) stimulation as in the pre-attentive MEG counterpart. The role of attention as an influence on neural processing in general indicates modulation of cluster excitability [29]. For auditory pattern processing, attention has effects on the MMN depending on how different deviant stimuli are perceived to be [20]. Deviants that are perceived to only differ slightly from a standard will be more affected than those that stand out [30], [31]. Considering the current use of complex pattern stimuli that span four to eight frequencies, we hypothesized that since deviants, which differed mainly by one frequency, will be more difficult to detect due to the small degree of stimulus change associated with them, differences between musicians and non-musicians under the influence of attention should be more pronounced.

## Methods

### Participants

Twelve musicians (8 female; mean age ± SD: 23.25±1.753 years) and 13 non-musicians (7 female; mean age ± SD: 27.33±2.60 years) participated in the study. Musicians were actively involved in practising music and had at least ten years of formal musical training on instruments and/or voice (11 to 18 years). Non-musicians had at most a very short period (<2 years) of musical training, and were not practising an instrument actively at the time of the experiment, and were not actively involved with music otherwise apart from simple recreational purposes (e.g. listening). Data of 3 participants had to be excluded (1 musician, 2 non-musicians) due to excess head movement (>0.5 mm) during MEG measurements. All participants were right-handed, as assessed by the German version of the Edinburgh Handedness Inventory [32], did not have any neurological or psychiatric conditions, did not have absolute pitch perception and did not deviate from average hearing capacities. All participants gave informed, written consent to participate in the study and received monetary compensation for their time and inconvenience. All procedures were conducted according to the Declaration of Helsinki and were approved by the ethics committee of the Medical faculty, University of Münster.

### Protocol

The experiment consisted of an MEG part and a following behavioural part. The MEG session consisted of four runs (three experimental conditions and a control condition) with short pauses in between, resulting in around one hour scanning time. In the three experimental conditions, a standard tone sequence of four, six or eight unique tones was presented repeatedly. Deviant sequences in which one tone of the pattern was changed were randomly interspersed as described in more detail in the stimuli section. The experimental runs took 9.43, 14.15 and 18.87 minutes of scanning time, respectively. The control condition of 8.33 minutes duration consisted of a classic auditory oddball paradigm in which only two different tones were used. This condition was included in order to obtain a reliable MMN in each subject. The order of the runs was balanced across subjects using a Latin square design.

After the MEG experiment, the test subjects participated in a shorter session of behavioural data acquisition of approximately 20 minutes duration. The setup of this session was identical to the one used in the experimental runs of the MEG experiment, except that fewer trials were included and participants were instructed to attend to the stimuli and gave a behavioural response whenever they noticed an unexpected tone that did not fit into the sequence. After the behavioural experiment was completed, the participants were asked if any sequence used in the experiment had sounded familiar to them. No participant reported that he had been reminded of a familiar piece of music by the tone sequences.

### Stimuli

All tones in the study had a length of 100 ms with rise and decay time of 10 ms. All tones were sine tones, as it has been shown that musicians show stronger evoked responses to the timbre of the instrument they are practising [33], which could inflate possible differences between musicians and non-musicians. The stimulation sequence of the control condition (standard oddball paradigm) consisted of 800 standard tones of 500 Hz and 200 deviant tones of 525 Hz, with a stimulus onset asynchrony (SOA) of 500 ms. In the experimental conditions, tones in the range of C3 to B4 of the Western diatonic C-Major musical scale were used. SOAs were 500 ms (+/− 25 ms jitter) in the MEG part, and 750 ms (+/− 25 ms jitter) in the behavioural part of the study, in order to stay within reasonable limits of scanning duration in MEG, and to allow for reaction times in the behavioural experiment. The slight jitter (on average only 5%) was introduced to prevent stimuli from occurring simultaneously and in-phase with scanner signal drifts and oscillations. During the MEG measurement, each experimental run consisted of 210 standard and 70 deviant tone patterns. In the behavioural measurement, 60 standard and 20 deviant patterns were presented in each condition, thus keeping the same ratio of standards and deviants.

**Figure 1:**
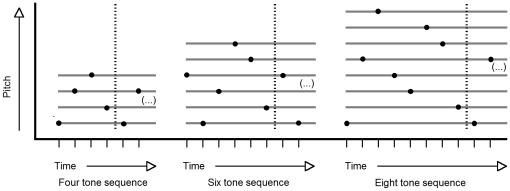
Examples of tone patterns for the three experimental conditions. Patterns were constructed individually from four, six or eight different tones, respectively, and the size and direction of intervals was controlled for between subjects. Time and pitch are indicated on arbitrary scales.

Standard patterns were constructed of unique tones such that no tone was repeated twice within the pattern (figure 1). In order to ascertain comparability across conditions, and to control the complexity and variability of melodic contours of the patterns across participants, we standardized the pattern construction process. The number of ascending and descending pitch intervals and the size and direction of these intervals in the sequences were balanced within and across conditions and subjects so that for a given sequence, no tone would be repeated twice, and the size of the interval between every pair of tones (eg. 1 and 2, 3 and 4, 5 and 6), would increase with increased pattern length. Due to this controlled randomization process, systematic differences regarding auditory grouping of tone clusters and harmonic expectancies within the patterns between conditions or groups were excluded. Also, the influence of contour on the generation of a representation [34] was restricted, balanced and comparable across all patterns and subjects. Although the tone patterns (but not pitches used in the patterns) were different for each subject, the same patterns were used for the MEG and behavioural parts.

**Figure 2:**
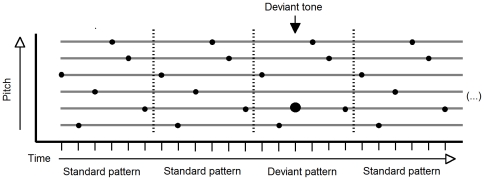
Example of three standard sequences and one deviant sequence as they might have occurred during the experiment. In deviant sequences one note at a random position (large dot) was shifted one tone up or down, unless this would be the same pitch as a neighbouring tone. Time and pitch are indicated on arbitrary scales.

The occurrence of deviant patterns within one run was randomized, with the restriction that at least one standard pattern had to occur between two deviant patterns (fig. 2). We decided to keep the ratio of deviant and standard patterns constant across conditions instead of fixing the overall ratio of standard tones and deviant tones by introducing more deviants in the longer patterns, because this would cause more variation per pattern and thus likely more strain on forming a coherent and constant representation. In other words, the number of deviants was comparable with respect to the underlying regularity (i.e. the pattern), not to the overall number of tones. Each deviant pattern was constructed such that one tone of a standard pattern was replaced by a tone that was one tone of the scale higher or lower than the original (and thus, all deviants were in key), while keeping within the boundaries of the pitch range of the pattern. If this meant that a tone would be repeated (due to an adjacent tone having the same pitch), the deviant tone was shifted one additional tone of the scale higher or lower. Note that such occurrences were balanced and evened out due to the randomization of all deviant positions. These restrictions were imposed in order to avoid deviants that would stand out because they were a tone repetition, or because they were outside the tonal range of the pattern. Although randomized, deviants occurred equally often in each position within a pattern within one run on average. In contrast to previous studies where the deviant occurred always in the same position of a tone pattern [10], [13], [35], [36], it is an important feature of the current study that deviants could occur in all positions of the patterns, because only in this case a detectable MMN response indicates that the complete pattern was encoded, whereas only part of the pattern would have to be encoded in detail if the deviant tone can be expected to occur in a specific position only.

### MEG data acquisition and analysis

For MEG data acquisition, a 275-channel whole-head system (Omega 275; CTF Systems Inc., Vancouver, Canada) in a silent and magnetically shielded room was used. Data were acquired at a sampling rate of 600 Hz. Participants were comfortably seated in the scanner, and their heads were fixed with pads inside the dewar. Head movement was monitored using three localization coils fixed to the nasion and both ear canal entrances. Alertness and compliance were monitored via video and audio recording in a direct link between the scanner room and the control room. Auditory stimuli were presented using sound transmission via plastic tubes at 60dB SL above the individual hearing threshold for each ear, which was determined with an accuracy of at least 5dB. Participants were instructed to ignore the auditory stimulation and to watch a self-selected silent movie which ensured their interest and attention to the distracting visual stimulation. Furthermore, they were asked to minimize head movement, eye blinking and swallowing.

For MEG data analysis epochs of 500 ms were extracted, with each epoch starting 100 ms before and ending 400 ms after tone onset. Trials with signal amplitudes larger than 3 pT were excluded from analysis. Standard tones preceding each deviant were used as standards in the analysis for all conditions. Standard and deviant trials were averaged for each condition for each participant. Averages were low-pass filtered at 30 Hz and high-pass filtered at 0.1 Hz. Then, averaged standard trials were subtracted from averaged deviant trials in order to extract the MMN field. For each condition, the MMN field was modelled by two spatiotemporal equivalent current dipoles (ECD), one in each hemisphere, in a spherical volume conductor based on the subject's head model, in a Cartesian based coordinate system. Individual head models were either based on the subject's T1-weighted MRI image or, if this was unavailable, on a Polhemus scan. In the control condition, dipoles were located within bounding boxes covering primary and secondary auditory cortices and residual variance was <15% in all subjects. Next, source waveforms were computed for averaging across subjects, in order to account for different head sizes and different positions of subjects within the dewar of the MEG scanner [37]. As it was not possible to identify the MMN and/or obtain a reliable ECD fit for the experimental conditions in all subjects, the ECD fitted on the MMN in the control condition was used to compute the source waveforms for all conditions. The source space projection technique is robust to slight displacements of the source, and similar approaches have successfully been applied in other studies where reliable MMN dipole fits were not obtainable for all subjects in all conditions (e.g., [7], [36]). From the source waveforms the average dipole moment within a 20 ms timeframe at the latency of the MMN peak (that fell within 110 to 220 ms) for each subject, hemisphere and condition were entered in a repeated measures ANOVA with factors group, hemisphere and condition. Also, in order to judge if the MMN amplitude in the different conditions and in both groups were significantly different from zero, a nonparametric bootstrap analysis (1000 iterations) was applied on the source waveforms.

### Behavioral data acquisition and analysis

For behavioural data acquisition, participants were instructed to press the left mouse button whenever they heard a tone that they felt or understood did not belong to the sequence of tones they perceived previously. Alertness and compliance was monitored directly by the experimenter who was present during the session. Participants were also seated comfortably and volume was adjusted so that stimuli were perceived as not too loud or soft. Responses were considered to belong to a tone if they occurred within the SOA, and within up to 200 ms after the next tone was presented, because responses within this time frame could not reasonably be assumed to belong to the succeeding tone. As a measure of performance, d-prime (computed as z[hit rate]- z[false alarm rate], where z is the z-transformed value, for hits to deviants and false alarms to standards) was computed. D-prime scores were then entered in a repeated measures ANOVA with factors group and condition. In all statistical analyses (MEG and behavioural), alpha was set at 0.05, and Greenhouse-Geisser correction was applied where appropriate.

## Results

### MEG Results

**Figure 3:**
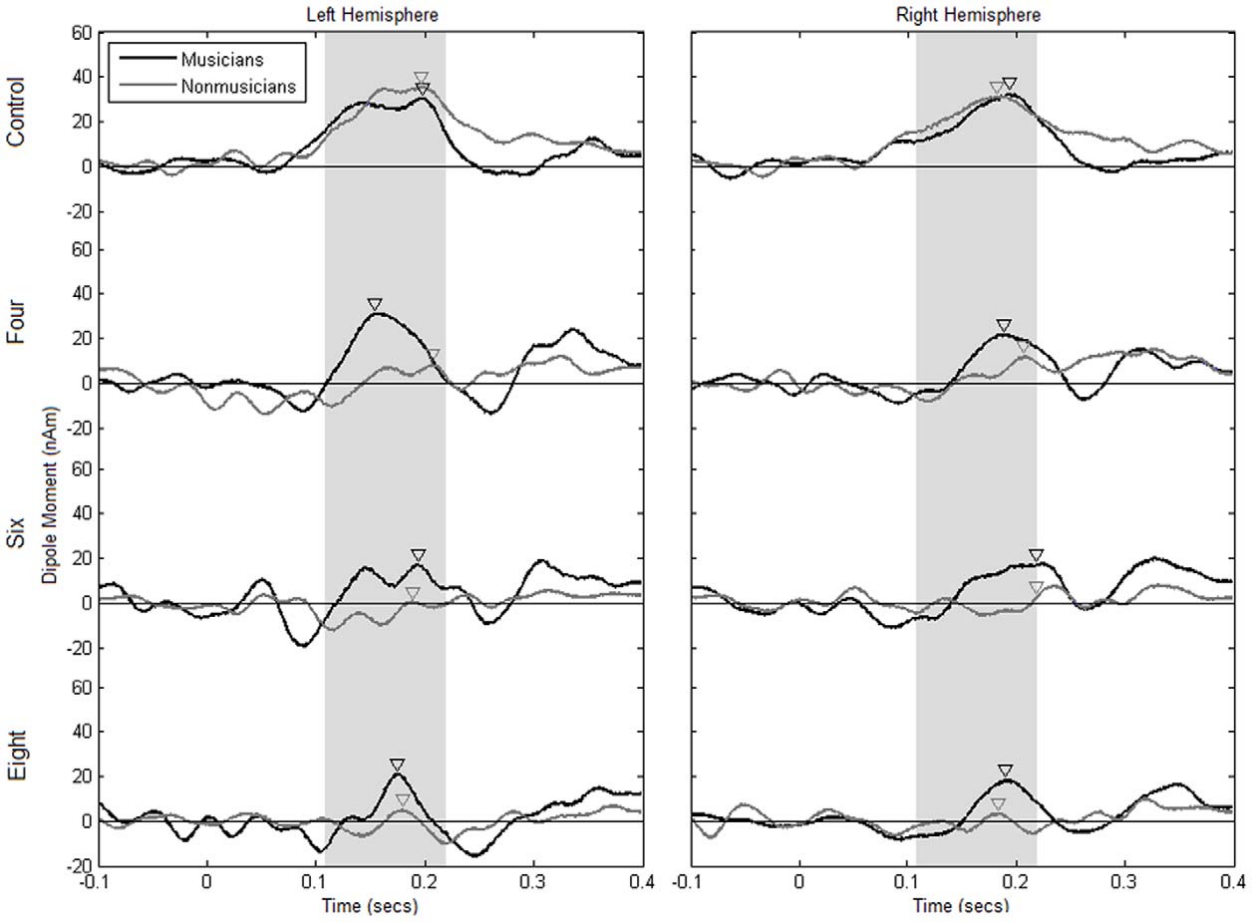
Group data (source waveforms) for the three experimental conditions (four, six and eight tone patterns) and the control condition and for both hemispheres hemisphere in the two groups (musicians  =  black traces, nonmusicians  =  grey traces). Arrowheads indicate the latency of the mismatch responses in the group averages.

**Figure 4:**
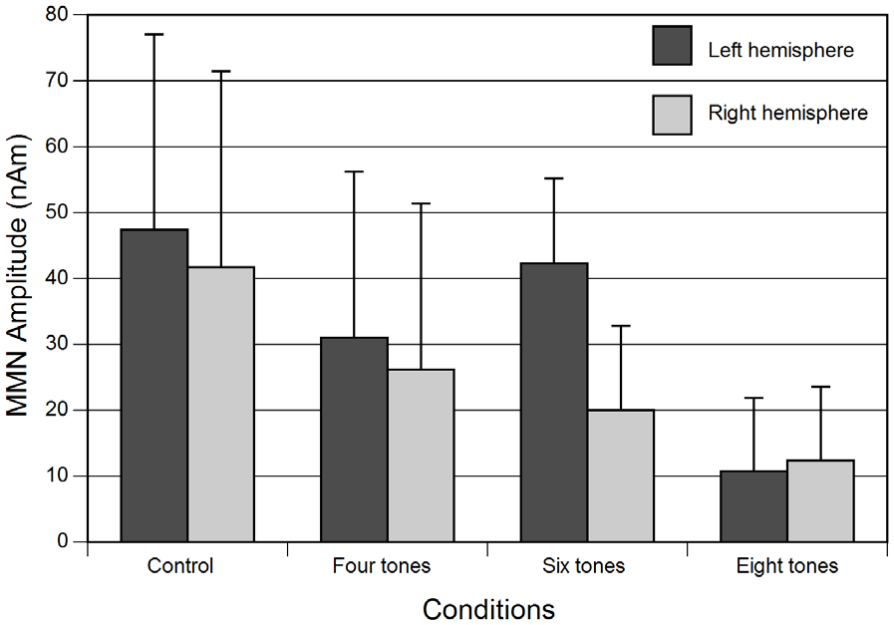
Mean MMN amplitudes for each condition and hemisphere for all subjects. The analysis of variance indicates a left-lateralized dominance for the six tone condition. Error bars represent standard deviations.

**Figure 5:**
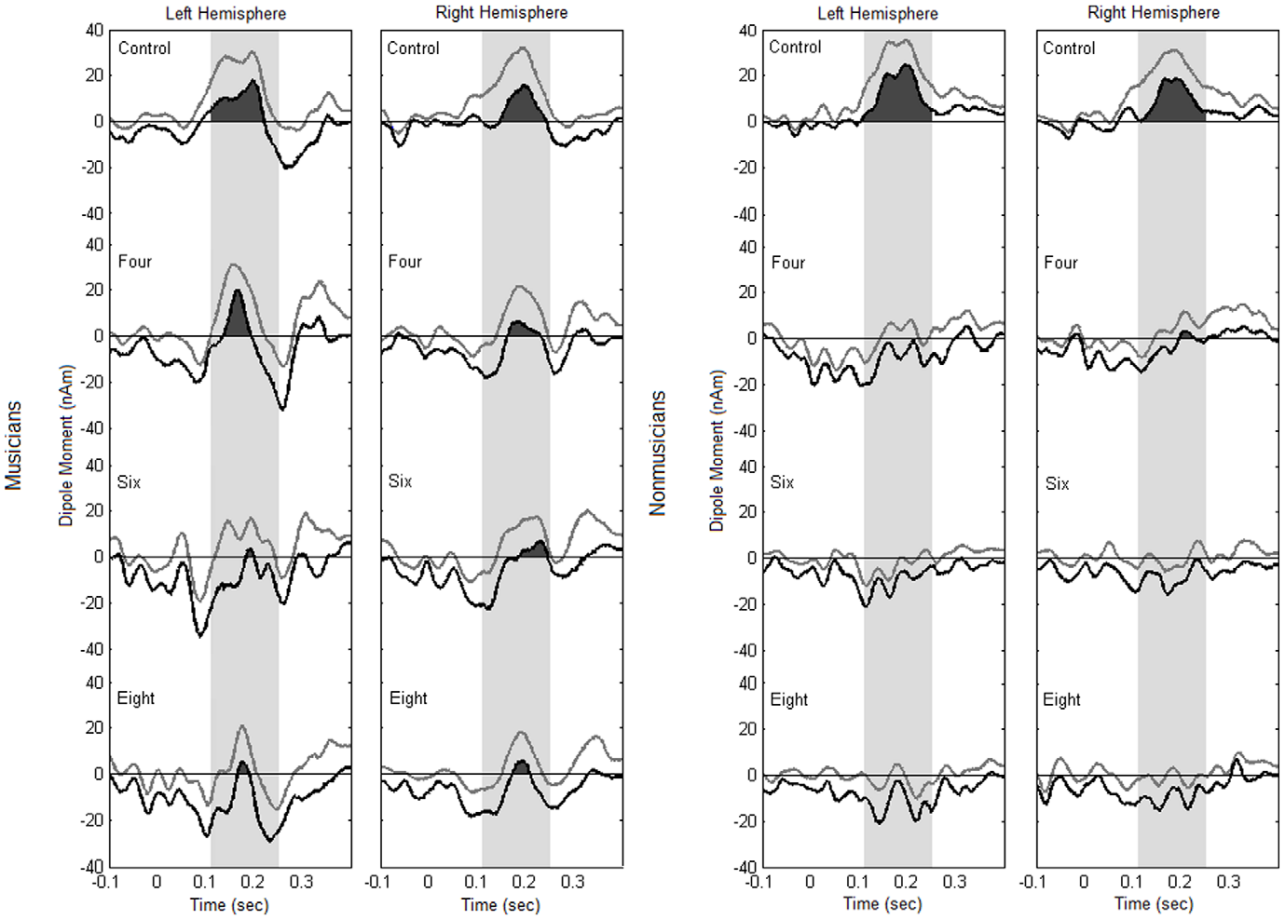
Lower boundaries (black traces) of generated bootstrapped confidence intervals for signal amplitudes (grey traces) for both groups in the three experimental conditions (four, six and eight tones) and the control condition for both hemispheres. Lower confidence intervals larger than zero indicate that there is significant deflection of source waveform. This is the case in all experimental conditions in both hemispheres for musicians, but only in the control condition, and in the four tone condition (right hemisphere) for non-musicians.

The repeated measures ANOVA on MMN amplitudes reveals that there is a highly significant difference among the conditions (main effect of condition, F(2,43) = 11.740, p<0.001), with the largest MMN in the control condition and decreasing amplitudes in the four, six and eight tone conditions. This can be seen in the source waveforms depicted in figure 3 as well as in the bar plot of the MMN amplitudes in figure 4. Planned contrasts show that there is a difference between control and four tone conditions (p = .007) as well as between the six and eight tone sequences (p<.001), but not between the four and six tone conditions (p = .890). Please note that the values of average MMN peak amplitudes do not match exactly in the source waveforms (figures 3 and 5) and in the bar plot (figure 4), because individual peak values are represented in figure 4, and because both groups have been averaged for this figure, as justified by an absence of an interaction of group, hemisphere and condition.

A main effect of hemisphere (F(1,20) = 7.889, p = .011) showed a left lateralized hemispheric preference of processing. This lateralization is most pronounced in the six tone condition, as visible in figure 4, which is supported by a significant interaction of condition and hemisphere (F(3,60) = 5.508, p = .002). Comparison of the amplitudes between hemispheres confirmed that in the six tone condition the MMN was significantly left-lateralized (pairwise t-test, t(21) = 4.092, p = .001), whereas there was no significant lateralization in the other conditions.

The ANOVA showed no significant difference between musicians and non-musicians (no significant main effects and no significant interactions involving the factor group). Although group average data of MMN source waveforms show a difference between musicians and non-musicians in the experimental conditions (see fig. 3), a large variance among participants possibly obscured between-group effects in the parametric statistical analysis. In order to deal with the high variance, nonparametric bootstrap analyses for each group and condition were run on the original source waveforms and confidence intervals (confidence interval of 0.95; zα = 1.96; p = 0.05) above zero were interpreted as significant effects. This is illustrated in figure 5, where the black areas below the lower confidence intervals indicate significant effects According to this analysis, both musicians and non-musicians show a strong MMN in the control condition, which justifies our approach to use the control condition dipole fit to compute the source waveforms. For non-musicians, there seems to be a very small effect in the four tone condition, and no significant MMN for the six and eight tone conditions. Musicians, however, show a significant MMN in all conditions in both hemispheres. Although this does not allow us to draw definite conclusions about differences between the groups, these data indicate a strong tendency towards a more pronounced and stable MMN in the musicians compared to nonmusicians, especially in the experimental conditions of six and eight tone sequences.

### Behavioral Results

For the behavioural data, d-prime was calculated. Group averages with standard deviations are given in table 1. In contrast to the MEG data, where no clear group effect or interaction involving group was observed, there was a significant effect of group in the behavioural data: musicians outperformed non-musicians greatly (main effect of group, F(1,20) = 47.949, p<.001). However, the interaction of group and condition was not significant, indicating that the difference between musicians and nonmusicians was not different depending on tone sequence length, but was comparable for all conditions.

**Table 1:** dPrime averages and standard deviations (in braces) for each condition and group.

dPrime averages	Four	Six	Eight
Musicians	3.28 (SD .304)	2.38 (SD .315)	2.01 (SD .568)
Nonmusicians	1.18 (SD .313)	0.39 (SD .456)	−0.30 (SD .343)

Similar to the MEG data, performance decreases as the tone patterns become longer. A main effect of condition (F(2,40) = 9.781, p<.001) confirmed that the task became increasingly difficult with longer sequences for both groups. Planned contrasts revealed that there was a difference between four and six tone conditions (four vs. six tone sequences: p = .013) whereas performance did not decrease significantly more from six to eight tone conditions (p = 0.75).

## Discussion

### Capacity of the memory store

Using a stimulation of repeated complex tone patterns of four, six or eight tones of unique frequencies, we investigated the capacity of the memory trace underlying the MMN response. Whereas previous research has mostly either focused on the decay of the memory trace over time [22], [23], [28], or on which kinds of patterns or other regularities can be encoded (e.g., [10], [12], [38]), to our knowledge no one has systematically studied the capacity of the memory trace in terms of the length of tone sequences, or in other words, the amount of information that can be stored in the memory trace underlying the MMN response. Our results show that both in musicians and nonmusicians the MMN was significantly different from zero in both hemispheres for the pattern of four tones. However, as the bootstrap results show, in the group of musicians even up to eight tones can be encoded into a representation on the basis of which deviant sounds can be detected, and which is precise enough to allow detection of subtle tone deviants. This can be concluded from the fact that a reliable MMN response can be observed to deviants that occur randomly in any position within patterns consisting of up to eight different tones.

Despite methodological differences, the present results might be related to estimates of short-term auditory memory capacity from behavioural studies. In a review on the literature, Cowan [39] concludes that the capacity of short-term memory for individual items of information (that cannot be chunked in a meaningful way) is most likely limited three to five items. This estimate fits well with the present results, where only for the four-tone pattern a reliable MMN response was observed in both groups, and indicates that short-term memory as assessed in behavioural studies might share similar mechanisms and neuronal correlates with the memory store underlying the MMN response. However, since contour complexity, deviant occurrence and tone pattern length covary in our stimulation, our estimate might understate the actual item capacity somewhat, since in addition to the extra pattern length for each subsequent condition, there are also additional pitches that need to be evaluated, while the deviant pitch displacement stays constant (in most cases one tone of the scale up or down). Also, these confounds might have added to the overall variance in the results. Therefore, it cannot be excluded that a short-term memory capacity of 6 to 7 items [40], [41] might also be possible for the memory store underlying the MMN response if simpler stimulus material is used. Future research on the encoding of complex patterns using stimuli of a wider pitch range could establish alternative methods to disentangle different factors that determine the capacity of the short-term memory in the context of the MMN literature and reconcile them with findings from other fields.

The main effect of frequency complexity and sequence length on MMN amplitude regardless of long-term expertise was significant. To our knowledge, no previous study has investigated this effect quantitatively. As the number of different tones increased simultaneously with increasing length (in order to create unique patterns without tonal repetitions), it is at this point not possible to distinguish the effects of both on the MMN. However, we can conclude that the MMN amplitude decreases with increasing demands on the memory store underlying the MMN response. As revealed by the planned contrasts in the analysis, while the difference between the four and six tone condition is not significant, the difference between the six and eight tone condition is. This suggests that, even though the absolute length of a sequence is increased linearly (by two tones additional tones between conditions), the MMN is affected in a non-linear fashion, which might indicate a qualitative change in processing within the corresponding cortical networks. One thing that must be considered due to the indirect nature of the MMN response as an indication for pattern encoding is that the absence of an MMN doesn't necessarily indicate that the pattern was *not* encoded. Possibly, despite a fundamental but rough (contour) representation of a pattern, it might not be encoded to a sufficient level of detail that would allow detecting deviants deviating by one tone of the scale only, especially if the deviant does not alter the contour of the tone sequence [36].

### Interpretation within the predictive coding framework

The present findings can be interpreted within the predictive coding framework on how neural representations underlying the MMN response are generated and constantly updated [42]. The predictive coding framework postulates that auditory input is continuously analysed and represented on different hierarchical levels. In the context of the current experiment, at first a pattern would not be represented as an actual pattern, but as a random sequence of tones, and as there are no repetitions of tones, no low-level representation of regularity (adaption hypothesis, [43]) would be formed – each new tone would be unpredictable on the basis of previous input. However, with more repetitions of the tone pattern, a higher-order model of the stimulus train representing the tone pattern will be formed and continuously adjusted and refined (model-adjustment hypothesis, [44]). When this higher-order representation is established, deviants can be detected based an incongruence of the input with this higher-order pattern representation and an according high-level prediction error, even if the prediction error on lower hierarchical levels is not different for standards and deviants due to large intervals and a number of different tones within the pattern.

It has been postulated that tonal input consists of two types of information that can be used in online sound stream analysis, a contour code and an interval code [36]. The contour code consists of information about the location and location changes of tones on the frequency spectrum and is predominantly processed in right hemispheric auditory cortical areas [45]. The interval code on the other hand is a more detailed representation of interrelationships between tones such as interval sizes. In the current experiment, only unique tones were used to construct each pattern and large intervals between successive tones were often present. Since deviant tones were shifted just one tone of the scale up or down, this possibly implied additional complexity due to a relatively high likelihood for such subtly deviant tones to not be detected and still be perceived as being part of the original sequence; in many cases, the contour of the sequence was not altered by the change of the tone, and importantly, all such changes were balanced due to the deviant randomization process. As it has been shown, changes related to the frequency interval are harder to detect than contour changes, especially for nonmusicians [36]. It is possible that, in terms of the predictive encoding model, higher order representations in some individuals do not contain such detailed information about sequences, but instead focus mostly on more global Gestalt characteristics [10]. The change-detection network might not be fine-tuned enough to regard subtle input changes during relatively fast stimulation as relevant enough to correct for in terms of future expectancy value, which could save cognitive computational capacity for other processes. Change-detection mechanisms in different individuals might have different thresholds for determining if something is unique input or not. We did not analyze contour and interval changes separately, due to insufficient signal to noise ratios. Future research could corroborate these arguments.

### The effect of musical expertise

It has frequently been shown that musicians compared to nonmusicians have superior processing capabilities in the auditory domain, especially for complex tone sequences (e.g., [7], [10], [12], [35]), and often physiological results are corroborated by behavioural data. In the present study, clear group differences in the behavioural task of detecting unexpected tones in the sequences were not reflected in significant group differences in the MMN amplitudes. Bootstrapped lower-boundary confidence intervals suggest that the MMN to pattern deviants is enhanced in musicians compared to non-musicians. However, in the parametric analysis, large within-group variance obscured any differences between musicians and nonmusicians.

It has previously been shown that large inter-individual variability in the MMN to complex stimuli can occur. Näätänen et al. [46] showed individual differences in the detection of deviance for complex sounds. In some individuals, a clear MMN was only visible after a longer series of repeated stimulus presentations, while in others it was detectable after only few. A possible reason for high variance within the musician group might lie in training emphases. Studying music professionally involves a lot of different cognitive abilities that are trained. Using short melodies, Seppänen et al. [47] found an earlier MMN response in musicians that used aural training strategies compared to the non-aural group for deviants that were interval changes, but not for contour changes. This notion is also supported by Tervaniemi et al. [35], who observed that training emphasis leads to different capabilities to discriminate changes in a melodic pattern. Subjects who are trained primarily without using a score (eg. jazz and pop musicians who improvise a lot), performed better than those who are trained primarily with a score, and this was also reflected in the MMN amplitudes to the deviants in the pattern. The current study did not control for the type of training musicians had received and the results of musicians versus nonmusicians might therefore be confounded by such training differences, especially since encoding not only the contour but also the interval structure of the tone patterns was important for detecting deviants. Also, different listening habits even among nonmusicians might influence their encoding on complex auditory information, and might be taken into account in further studies. Interestingly, considering the bootstrapped lower confidence interval results of the current study (fig. 5), there does seem to be an indication that at least a majority of musicians have improved aural discrimination capacities.

### The effect of attention

In the current study, a highly significant group effect for the behavioural measure was found, which indicates a clear difference between musicians' and non-musicians' abilities to detect deviants embedded in a complex tone pattern when attending to auditory input. This is in line with findings of Tervaniemi et al. [48] who found that musicians have enhanced processing of sound (and speech) patterns during attentional listening compared to non-musicians. Cowan [39] also concludes that the capacity of auditory short-term memory can be expanded by attention and elaboration. Our data are in line with a model where tone patterns are encoded in an auditory short-term memory store that has a limited capacity regarding the number of items that can be maintained (around 4 tones in most individuals) and that serves as a reference for pre-attentive change detection (cf. the predictive coding model) and the capacity of which seems largely unaffected by long-term musical training, whereas auditory short-term memory under attention, enabling more detailed analysis of sound patterns, seems to be enhanced by long-term musical training. The complexity of the stimuli used in this study most likely required the influence of attention to be analyzed in sufficient detail. Note that such differences are not confounded by sequence complexity or deviant occurrence, since these were identical for musicians and non-musicians. Also, whereas the main difference between conditions in the MEG results was between the six and eight tone conditions, these conditions did not differ significantly in the behavioural data, where the main difference was already between the four and six tone conditions. This suggests that the auditory memory underlying the MMN response versus behavioural performance is differently affected by increasing difficulty or stimulus complexity.

### Lateralization

A further point worth discussing is the left lateralized activity pattern in the MEG data. Left hemispheric auditory processing is associated with temporal cues [49]. We have previously reported a left-lateralized pattern MMN in a setup where deviants differed in the temporal domain (number of tones preceding a pitch change) rather than in the pitch domain [7]. In the current study we found that a left-lateralization was only present in the six tone condition. One possible explanation for this might be the fact that sequences in the four and eight tone conditions can be grouped according to a standard 4/4 time signature, the meter that would be associated with the six tone sequences (3/4, or waltzing meter) is different from the other two. Given the more common occurrence of the 4/4 meter in classical and popular music, the ¾ ‘meter’ of the three-tone sequence might be considered a more salient temporal structure in the context of this experiment, leading to an additional recruitment of networks that process patterns in the temporal domain and that are predominantly located in the left hemisphere [7], [50]. However, in contrast to Vuust et al. [51], this lateralization was not modulated by musical expertise in the present study. Left hemispheric activity is often associated with language processing, mainly because language is associated with rapid vocalizations and short transitions in pronunciation (e.g., [52]). However, this notion has been challenged on several occasions. For example, some studies seem to find predominantly right hemispheric activity in response to duration deviants (e.g., [53], [54]). The current study is an important indication that the reverse can also occur, in that a pitch deviant can elicit predominantly left hemispheric activity for complex tonal input.

### Conclusion

The results of the present study indicate that the capacity of the memory trace underlying the MMN is in most persons limited to sequences of around four different tones, similar to estimates of the capacity of short-term memory. The data also suggest that musical training enhances this capacity during attention to the stimuli more so than during pre-attentive processing, and that other factors such as the metric structure of the tonal patterns influence the neuronal encoding. The results are in line with recent models of adaptation and prediction mechanisms in auditory cortex and add to our understanding of the neuronal basis of auditory short-term memory.
